# Supplementary material for: SMORE: spatial motifs reveal patterns in cellular architecture of complex tissues
Source: Genome Biol. 2025 Jan 3;26:3. doi: 10.1186/s13059-024-03467-5 (PMC11697875; doi:10.1186/s13059-024-03467-5)
Supplement: Supplementary file 1 — Additional file 1: Supplementary Material for SMORE: spatial motifs reveal patterns in cellular architecture of complex tissues. This file contains recommendations for handling large sample numbers, side-by-side comparison of SMORE with HistoCAT and ImaCytE’s performance on the retinal bipolar dataset, and analysis of a whole mouse brain spatial transcriptomics atlas, as well as Figs. S1 to S7 and Table S1. [file 13059_2024_3467_MOESM1_ESM.pdf]

Supplementary Material for SMORE: spatial motifs reveal patterns in cellular architecture of complex tissues

Zainalabedin Samadi[1], Kai Hao[1], Amjad Askary[1]

[1]Department of Molecular, Cell and Developmental Biology, University of California, Los Angeles, Los Angeles, 90095, CA, USA.

Contributing authors: zainsamadi@ucla.edu; kaihao@ucla.edu; amjada@ucla.edu;

Supplementary Information

Recommendations for Handling Large Sample Numbers

Given that URPEN sampling is uniform, even with a low sampling frequency, it is possible to obtain a reliable estimate of the total number of paths in the graph. This allows users to adjust the sampling frequency as needed without compromising accuracy. Generally, the default settings of the method work well for typical datasets. However, for larger datasets (e.g., those with more than 5 million path samples), the following recommendations can help balance performance and computational cost:

1. Start without Enrichment: For large sample numbers, consider beginning analysis without applying enrichment. This reduces computational demand and allows for an initial exploration of patterns.
2. Lower nTrain and nScore Values:
   (a) nScore affects only the ordering of output motifs, not their structural content. Therefore, it can be set to a minimal value (e.g., nScore= 1) if output order is not critical to your analysis.
   (b) nTrain impacts the number of control datasets generated to identify significant motifs. While larger nTrain values yield more robust results, they are not always necessary for large datasets with fewer cell types, since there are enough cells to compute significance reliably. For such cases, a lower nTrain value may suffice.
3. SMORE Default Settings: The default settings for SMORE involve fully sampling length-4 radial paths of the input graph, with nTrain= 50 and nScore= 10. For very large datasets, these values can be adjusted to optimize performance without sacrificing analytical accuracy.

Side-by-side comparison of SMORE with HistoCAT and ImaCytE's performance on the retinal bipolar dataset

We applied two methods for identifying spatial neighborhoods, HistoCAT [8] and ImaCyte [19], to our bipolar dataset for comparison with SMORE's outputs. Each method offers unique strengths and limitations, which we evaluated by examining similarities and differences in the motifs they detect.

HistoCAT identifies pairwise interactions, producing outputs that correspond to SMORE's length-2 motifs. However, HistoCAT does not identify higher-order motifs, i.e., arrangements of more than two cell

types. To highlight this distinction, in addition to SMORE's length-2 results (Fig. S4a), we included its length-3 motifs (Fig. S4b). SMORE's length-2 motifs are presented as a heatmap to facilitate comparison with HistoCAT output.

While both HistoCAT and SMORE identify pairwise interactions, they differ in their approach to statistical significance. HistoCAT assesses significance based on the proportion of tissue samples in which a motif is observed, using a permutation test with a threshold such as $p \leq 0.05$. SMORE, by contrast, calculates p-values from ZNIC counts across all tissues, using a negative binomial test. An example of difference in length-2 motifs between SMORE and HistoCAT includes the "ON" motif (involving an RBC and a BC9 cell type) (Fig. S4a, c). While HistoCAT identifies this motif as significant, SMORE does not. In the input dataset, there are 787 instances of the "ON" motif, yielding 310 ZNIC sites, with an average of 2.52 interactions per site (787 "ON" samples divided by 312 unique "N" neighbors). In contrast, a control dataset includes 817 "ON" samples and 371 ZNIC sites, averaging about 2.2 interactions per site ($\frac{817}{374}$). SMORE does not identify this as a motif because the original dataset has fewer ZNIC sites (310) than the control (371), suggesting a lack of enrichment. HistoCAT, however, detects it as significant because the average number of "N" neighbors for "O" cells in the original data (2.5) is higher than in the control (2.2). It should be noted that HistoCAT analyzes samples independently, so the interaction rates provided here are approximations of the HistoCAT algorithm's broader process. Interestingly, if we fix the "O" cells in SMORE, the "ON" motif does become significant, with the average ZNIC site count in control data dropping to 231 across 50 control datasets. SMORE also identifies certain motifs, such as "LE" and "LF", as significant that HistoCAT does not detect, highlighting differences in how these methods capture specific spatial patterns.

ImaCytE, on the other hand, identifies significant microenvironments or niches within the tissue without specifying a motif length. In its output in Fig. S4d, ImaCytE detected a motif involving an RBC cell with Type 2 (C) and Type 6 (K) cells in its neighborhood—similar to our most significant COOK motif, which we hypothesize is associated with scotopic (low-light) vision. However, ImaCytE does not specify the sequence of cell types in each motif, which can be crucial in defining functional motifs. For instance, the structure of the scotopic pathway illustrated in Fig. 4d aligns well with a motif like COOK, where an RBC cell is central, flanked by ON and OFF bipolar cells on either side, capturing the spatial relationships relevant to this visual process. Further, ImaCyte output doesn't involve a motif resembling HBBI, a motif that based on our gene expression analysis, can explain the mode of action of type 1b OFF bipolar cells in the retina.

In summary, while HistoCAT and ImaCyte detect spatial neighborhoods or cell arrangements, they each have limitations: HistoCAT does not capture motifs longer than two cell types, and ImaCyte does not account for cell sequence within motifs. SMORE 's ability to identify both pairwise and higher-order motifs, along with its focus on sequential order of cell arrangements, allows it to reveal biologically meaningful patterns that are overlooked by these methods.

Scalability of SMORE: Analysis of a whole mouse brain spatial transcriptomics Atlas

To demonstrate the scalability of our spatial motif analysis approach, we applied SMORE to the Allen Brain Cell Atlas of a whole mouse brain [50], which includes approximately 4 million cells across more than 5,200 cell types. A 20% sampling of the neighborhood graph from this dataset generated over 27 million samples. SMORE successfully processed this data volume without enrichment, completing the analysis in approximately 15 hours. We labeled cell types based on their frequency, with the first 52 most frequent cell types assigned letters, starting with upper case and ending with lower case letters in alphabetical order (e.g., "A" for the highest frequency and "z" for the lowest within this subset). The remaining cell types were labeled with random characters. This labeling allowed us to capture motifs composed of both common and less frequent cell types, showcasing SMORE's capacity to handle large-scale, complex datasets (Fig. S7).

Supplementary figures

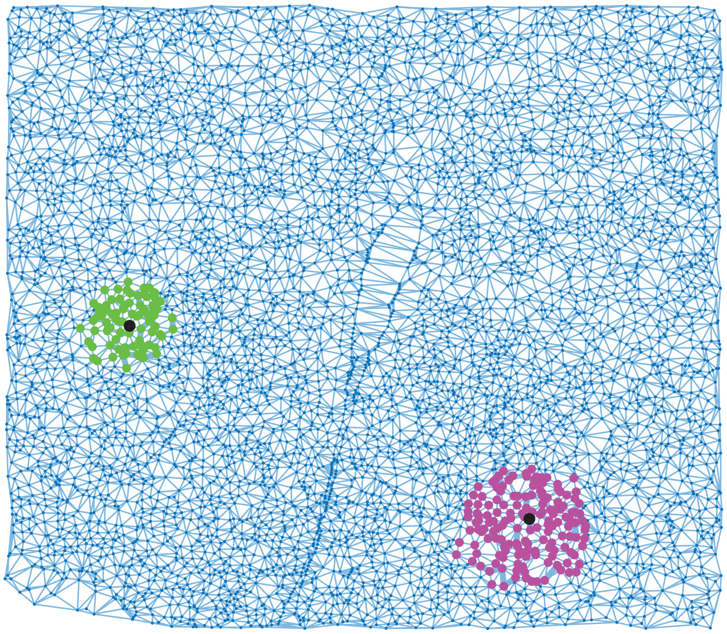

Fig. S1*:** ***Examples of kernel neighborhoods. *An example of the kernel around the center black colored node from the mouse hypothalamic preoptic region dataset. Highlighted nodes in green are length* 4 *kernel nodes and magenta-colored nodes are length* 6 *kernel nodes.*

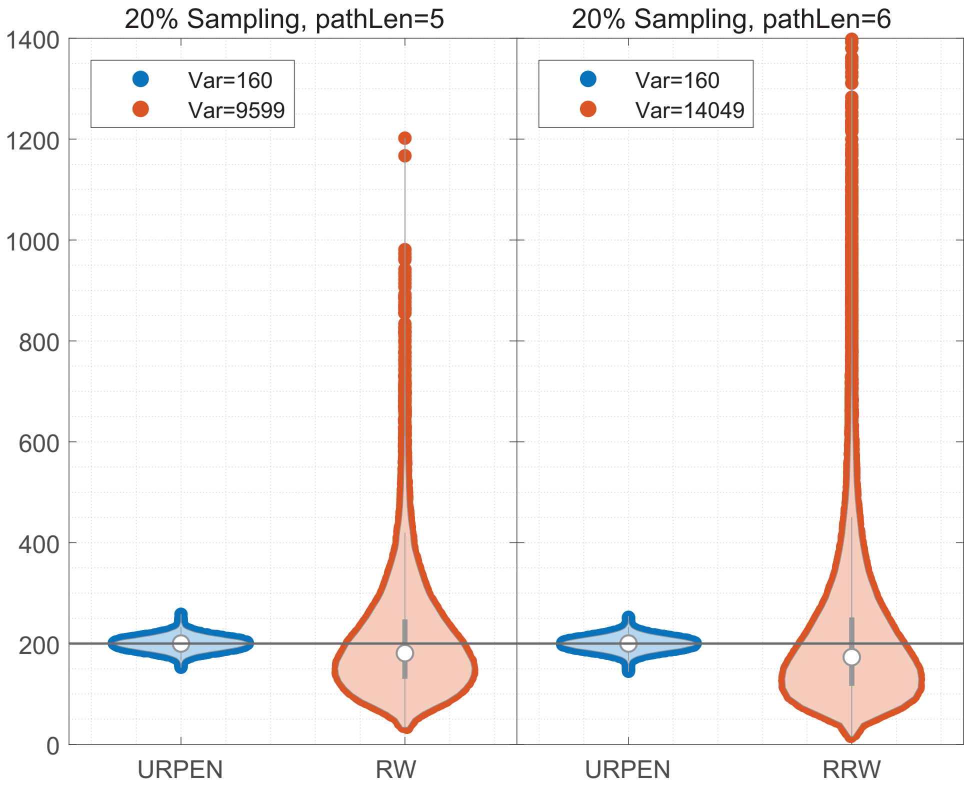

Fig. S2: ***Unbiased sampling of paths from neighborhood graphs.*** *URPEN and random walk (RW) is used to sample* 20% *of the graph described in Fig. 2 for length* 5 *non-radial samples and length* 6 *radial samples. In both cases, URPEN samples paths uniformly, while random walk is not uniform. The sampling probability in URPEN is set to* $p = (1, 1, \ldots, 1, 0.2)$ *and the test was performed* 1000 *times.*

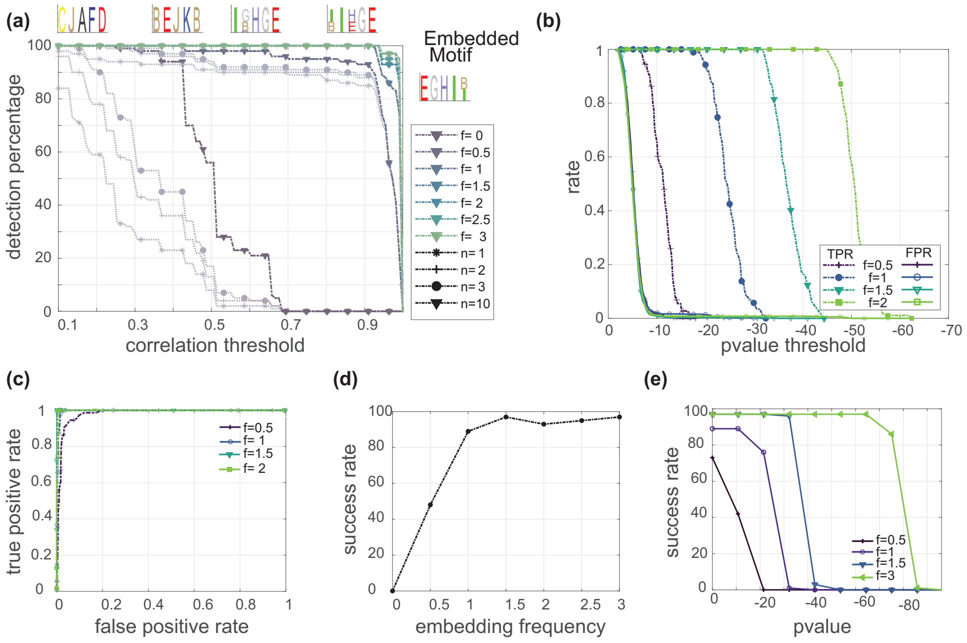

Fig. S3: ***Evaluation of SMORE's performance on synthetic data with known ground truth.*** *Evaluation of SMORE's performance on synthetic data with known ground truth. Same as Fig. 3, for length 5 motif.*

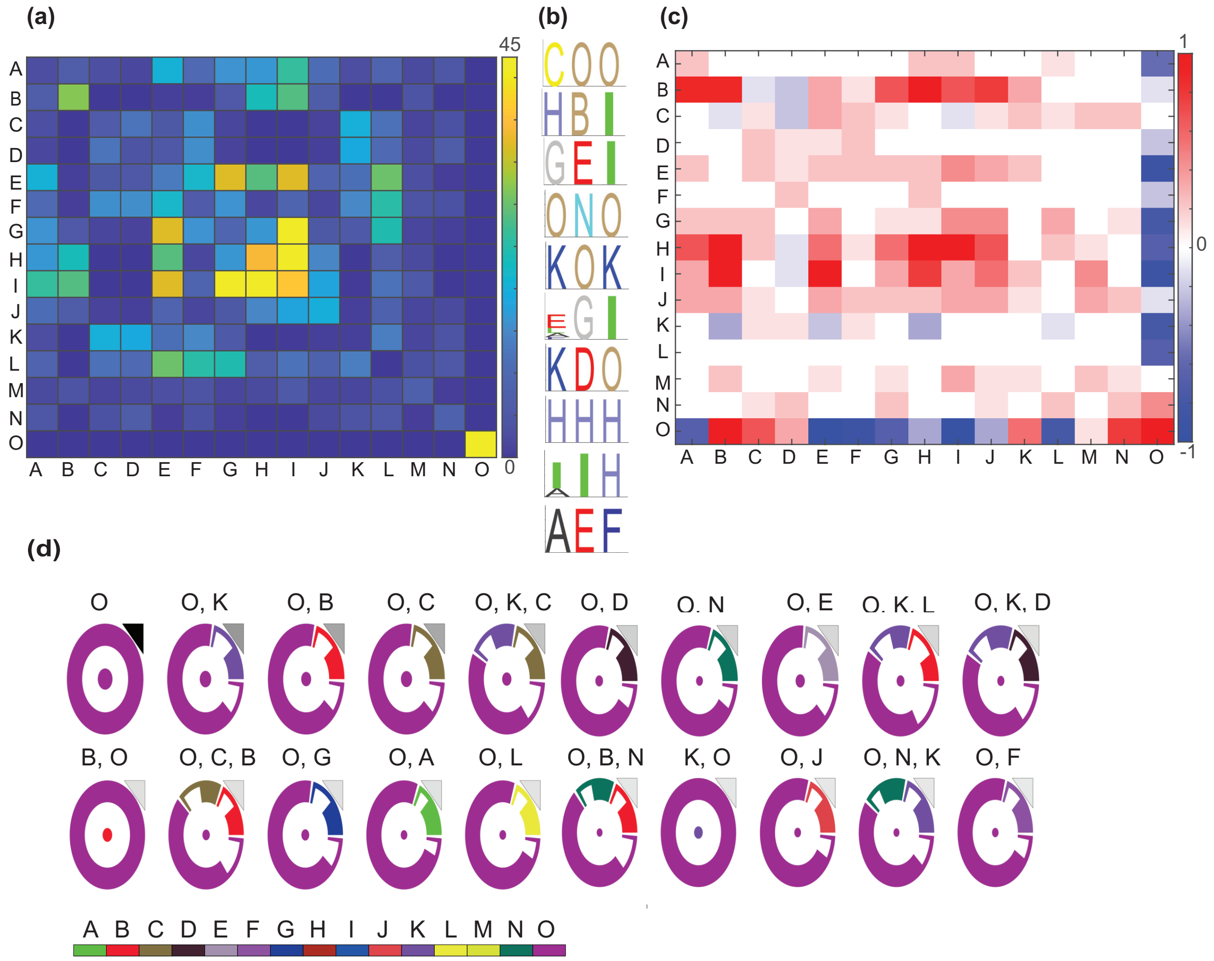

Fig. S4: ***Side-by-side comparison of SMORE, HistoCAT, and ImaCytE performance on the retinal bipolar dataset.*** ***(a)*** *SMORE length-2 output result on the retinal bipolar dataset.* ***(b)*** *The top 10 SMORE length-3 motifs with RBC cells fixed.* ***(c)*** *HistoCAT's output results on the retina bipolar dataset.* ***(d)*** *ImaCytE first 20 output motifs ordered by z-score, from top left to bottom right. Colors representing each cell type are shown at the bottom.*

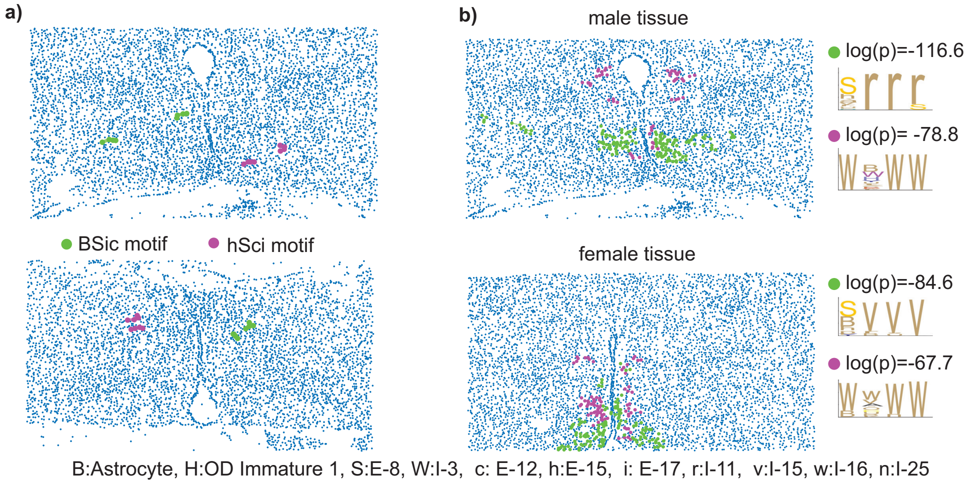

Fig. S5**:* ***Highlights of the specific motifs in mouse hypothalamic preoptic region. (a) *Highlight of two similar motifs in male tissues, animal IDs, 10, and 11. Involved cell types are B: Astrocyte, S: E-8, c: E-12, h: E-15, i: E-17.* ***(b)*** *Two similar motifs in male and female tissues. First motif in male tissue mostly consisted of (S: E-8, h: E-15, á: I-34) in the first position and r: I-11 in the remaining ones. For the female tissue, the first motif mostly consists of (S: E-8, h: E-15, p: E-23, Y: I-7) in the first position and v: I-15 in the remaining positions. The second motif which mostly consists of w: I-16 for both tissues, involves (W: I-3, Y: I-7, t: I-13) in the second position of the male motif and (w: I-16, v: I-15, Õ: I-10) in the second position of the female motif.*

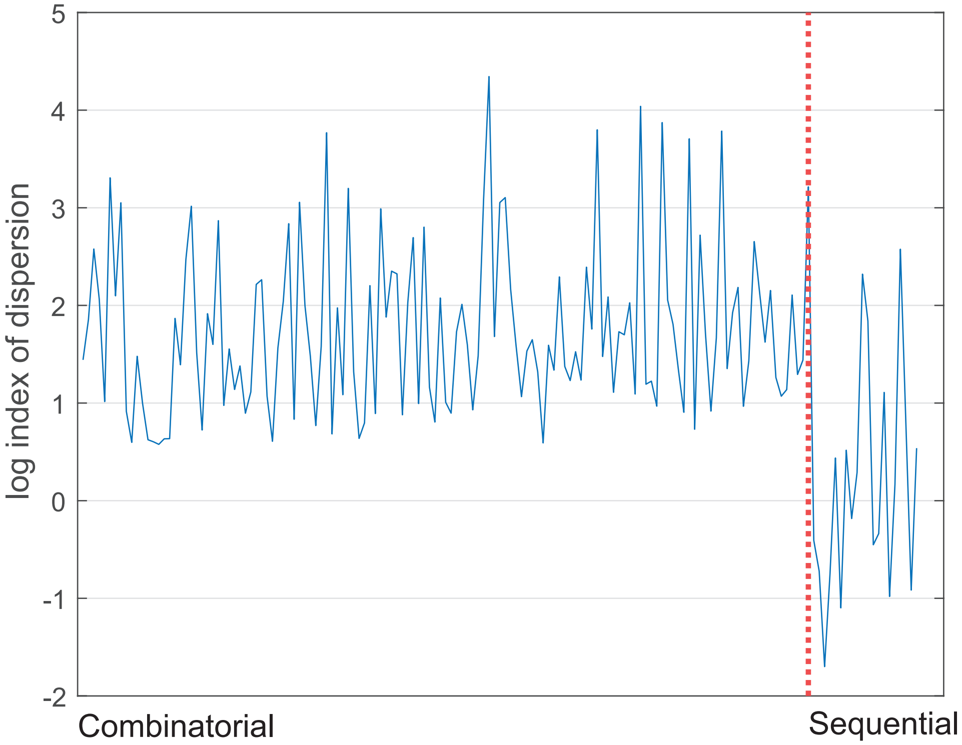

Fig. S6**:* ***Index of dispersion for combinatorial and sequential genes. *Index of dispersion for gene expression values obtained through combinatorial MERFISH and sequential smFISH. This coefficient is defined as the ratio of variance over mean. Negative log values (index of dispersion less than 1) are under dispersed. All combinatorial genes are over dispersed, while most sequential ones are under dispersed.*

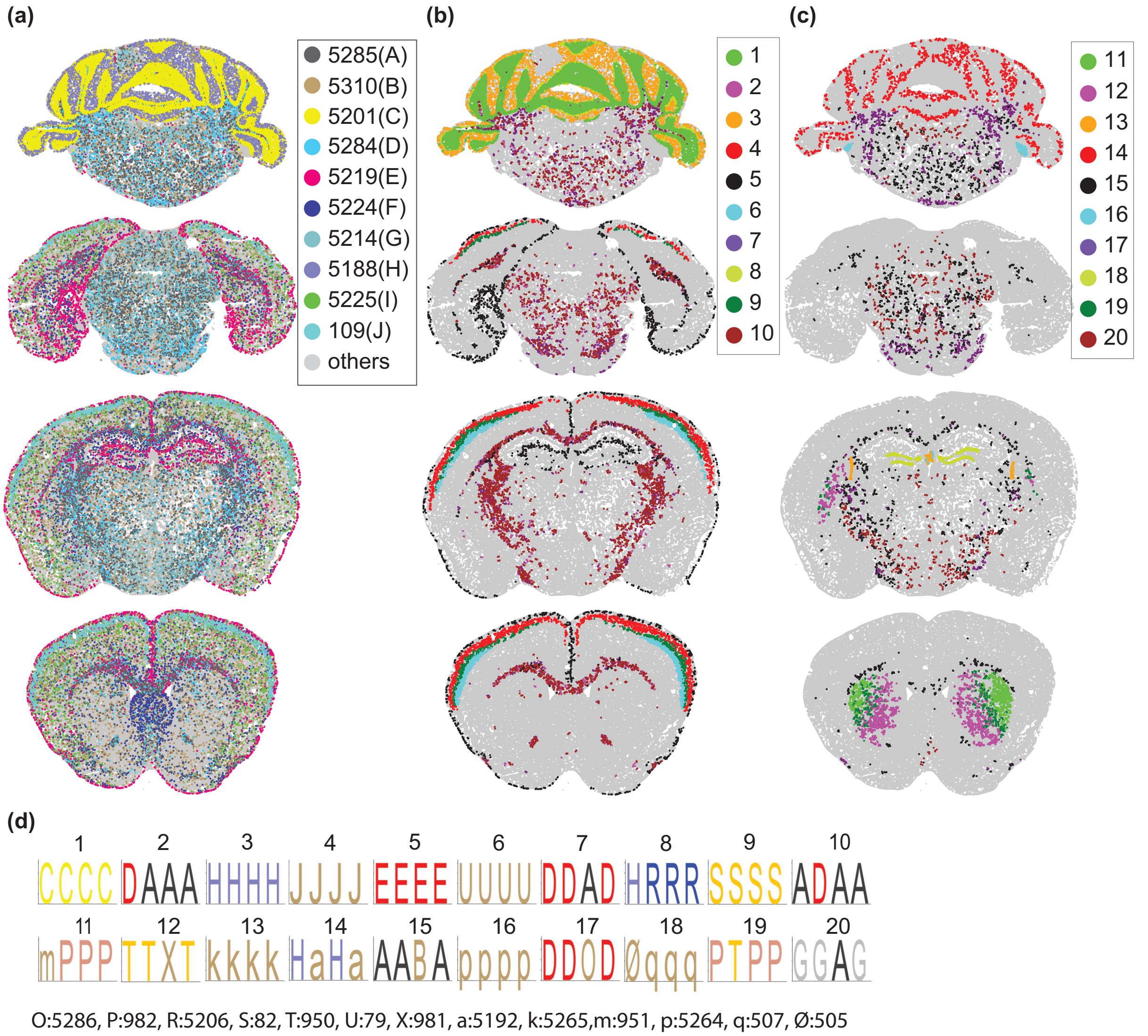

Fig. S7**: **Spatial motif analysis of a whole mouse brain spatial atlas. ***(a)*** *Neighborhood graphs of four example sagittal sections. The method is applied on the entire 59 sagittal sections in the dataset. Four sections are selected here to facilitate illustration.* ***(b)****First 10 motifs obtained using global shuffling to generate the control data along with their highlighted nodes on the tissue graph. Each motif is indicated by a different color in the highlighted tissue graph.* ***(c)*** *Same as (b), second 10 motifs.* ***(d)*** *motif logos for the first 20 motifs. Annotations for the cell types involved in the motifs are either listed in the legend for the panel (a) or at the bottom of the panel (d). Detailed description of cell type cluster IDs are provided in the reference paper [50].*

Supplementary tables

***Table S1**: The timing performance for the experimental datasets processed in this study. System Specifications: Processor Intel(R) Core(TM) i9-10900X CPU* 3.70 *GHz, Installed RAM* 64.0 *GB. The settings for all datasets are the same with sampling frequency of* 1 *and nTrain=* 50*, nScore=* 10*, except that sampling frequency for 3D hypothalamus data is* 0.1*, and nTrain for Mouse Embryo dataset is* 10*. W refers to the desired motif length.*

dataset	Number of cells	Number of samples	URPEN required time	SMORE required time (seconds per motif)	Gene Expression analysis (seconds per motif)
Bipolar (W=4)	26946	524,259	23	1000	< 60
Bipolar (W=5)	26946	1,143,575	67	3111	< 60
Hypothalamus (W=4)	28866	696,141	24	1816	< 60
Hypothalamus (W=5)	28866	1,740,501	75	5724	< 60
3D hypothalamus (W=4)	78229	1,838,093	192	4228	NA
Mouse Embryo (W=4)	256486	6,190,540	203	3288	4300
