# Supplementary material for: SMORE: spatial motifs reveal patterns in cellular architecture of complex tissues
Source: Genome Biol. 2025 Jan 3;26:3. doi: 10.1186/s13059-024-03467-5 (PMC11697875; doi:10.1186/s13059-024-03467-5)
Supplement: Supplementary file 2 — Additional file 2: Review history. [file 13059_2024_3467_MOESM2_ESM.docx]

**1^st^ round**

**Reviewer 1**

The authors introduce a new approach to detect spatial motifs, representing recurring cell arrangements in tissues. Using synthetic and real data, the authors showcase the accuracy of their method in identifying these motifs, thereby unveiling novel insights into cell organization and gene expression in neural tissues. This approach has the potential to unveil tissue complexity, provide novel perspectives even in well-studied models, and generate testable hypotheses.

However, I have some concerns regarding the description and presentation of data in the manuscript.

Major concerns:

1. The manuscript lacks a detailed description of existing methods. In the Introduction section, the authors mentioned: "Therefore, existing methods for network motif analysis that focus on patterns of interactions between the nodes, rather than the identity of the nodes themselves, are not suitable for analysis of spatial data." In the Abstract section, the authors state: "most existing methods for analysis of spatial data only examine distribution of one or two labels at a time." However, the manuscript lacks detailed descriptions and examples of these "existing methods". It should provide comprehensive explanations of existing approaches, including examples, along with articulating the advantages of the new method over existing approaches.

2. The advantages of the new method need to be further refined and substantiated. Indeed, whether the motifs discovered by this method are not identifiable by other approaches (such as those identifying niches in spatial data), requires further substantiation with data. Moreover, concise descriptions of the method's advantages over others and its biological value should be refined and included in the Introduction and Abstract sections.

3. The applicability to various tissues and cell types needs to be clarified. The manuscript primarily demonstrates the application of the method to spatial datasets composed mainly of neural cells (mouse retina and hypothalamus). Further clarification is needed regarding whether the method remains applicable to spatial data from other systems and cell types.

4. The applicability to various numbers of cell types needs to be clarified. The two datasets presented in this manuscript are from the mouse retina and hypothalamus, comprising over 100 and over 70 cell types, respectively. However, many spatial datasets do not contain as many cell types. In cases where there are fewer cell types, such as 10+ to 20+, does this method still apply? Further clarification is needed.

5. Further clarification is necessary regarding the application of this method on other sequencing platforms. The authors mentions in the Introduction section:"In contrast, new spatial techniques can profile the expression of many genes, or even the entire transcriptome. In this way, the position of many cell types can be mapped in the same sample. However, this feature is not directly utilized in virtually any of the existing analysis method…" All spatial transcriptomics data used in the manuscript are from -FISH sequencing platforms (SABER-FISH and MERSH), which have fewer genes compared to 10x Visium and Slide-seq technologies. Can this method be applied to 10x Visium and Slide-seq data? Additionally, further explanation is needed on how this method demonstrates the utilization of more genes.

6. Figure 3 demonstrates the performance on simulated data. However, how was this simulated data generated, and how overrepresented motifs were added? This process requires further clarification.

7. The manuscript lacks detailed descriptions of the methodology. For instance, in section 2.1.1, the author mentions: "…though this requirement is not necessary for directed graphs." The author utilizes Delaunay triangularization to construct a spatial graph, but how are directed graphs generated, and how is the direction determined? This may confuse readers. Similarly, the definition, significance, and references of ZNIC model and ZNIC counts should be further clarified.

8. In Figure 2d, the author evaluates the efficiency for k=4-8. However, in subsequent practical applications, the author mostly uses k=3. Therefore, the evaluation of efficiency in Figure 2 may not accurately reflect the real computational cost. It would be beneficial if the author could assess the computational cost on real data, including time and memory, as these are important considerations for users in practical usage.

9. What impact do different choices of k have on performance and computational cost? Can the author assess this and provide recommendations to users?

Minor concerns:

1. In Figure 1a & 1c, please label the cell types for better reader comprehension.

2. In section 2.2, the author describes STREME as "…which the author has demonstrated to be more accurate, sensitive, and comprehensive than several widely used motif discovery algorithms." The task in this manuscript differs from that of the STREME article. Regarding whether STREME is also applicable to this task, could the manuscript provide some discussion? Additionally, STREME has only one author, so it should not be referred to using the plural "authors."

**Reviewer 2**

The authors propose a brand new concept of spatial motif, which is a very novel work that can help us to parse spatial transcriptome data from more perspectives. There are several problems to be solved by the authors as follows:
1. Please specify the "f" in figure 3 caption.
2. Please explain clearly the correspondence between "spatial motif" and "motif #1-5" in figure 6d/e.
3. Due to unclear figure captions, it is difficult to fully grasp the author's intentions in figures 4b/c and figures 6d/e. Please provide additional details to complete the figure captions.
4. Please elaborate on the differences between spatial motifs and the actual spatial locations of cell types. What are the advantages of using the spatial motifs for characterizing tissues?

**Reviewer 3**

The paper in itself is of interest, and the modeling of spatial configurations is the to go for this type of data; However, though the concept is powerful, and certainly of interest to the community, I have a number of major concerns:
-I miss a number of highly relevant prior works that already did spatial cell motif modeling before spatial transcriptomics came about as a relatively new kid on the block. For instance, in Imaging Mass Cytometry, detailed cell type annotation followed by spatial configation modeling is commonplace for about a decade, and VeTRA and CODEX immunofluorescence tissue imaging idem dito. Software like histoCAT (Raul Catena), ImaCyte (Somarakis), and SpaceCo (again Somarakis) were specifically designed for detecting overrepresentation of cellular motifs in cohort comparison studies, and I think the related work should be reworked to include cell motif modeling in tissue regardless of the readout technology (spatial transcriptomics, Imaging Cytof or Imaging Mass Spectrometry). The presented approach in the current paper has indeed novel elements, but it is now presented as a first to look at complete cellular arrangements, and this claim should be toned down a lot. Original element I find the deployment of a motif discovery from genome sequences for detection of cellular motif overrepresentation, and that in itself is innovative enough.
-I find it difficult to distill the exact goal of the motif discovery: identify spatial motifs, but for what? Is the exact goal to find morif descriptors for a piece of tissue, or is it geared towards finding differences between patient groups (e.g. responders vs non-responders). This overarching goal should be articulated much more clearly.
-After introducing the necessity of motif discovery, I miss the exact motivation of the methodology that follows, and it read a bit like "let's go mine graphs with neighborhood info encoded". I totally support the presented method, but a bit more explicit motivation of the choice of methods would be beneficial for the paper clarity. Having said that, I find the method for sampling the graph original, and also subseqient motif discovery.
-I find the validation convincing, with first motif detection of artificially introduced motifs, and then a number of interesting real data findings. I also find the comparison of gene expression of cells of a specific type that are part of a motif in comparison to the same cell type not part of a motif highly informative. It would be interesting though to investigate whether the same motifs are recovered by HistoCAT of ImaCyte; with relatively minor effort this should be feasible.
Final question: perhaps the authors can discuss the scalability to millions of cells: for instance the ABC-Atlas of spatial transcriptomics of a 3D whole mouse brain; Could this method be developed to develop a "spatial motif atlas of the whole brain?"
Taken together, I find that this paper can certainly become of broad interest to others in the field, by addressing a number of the concerns I voiced.
Though search for motif overrepresentation is not novel, the way that the authors address this is novel indeed.

**Authors’ response**

We appreciate the thoughtful and constructive comments from all three reviewers. In this revision, we addressed all reviewer comments with addition of new analysis and results as well as changes to the text, figures, and figure captions. The most significant changes include:

- **Positioning our new method in relation to existing approaches.** We heavily revised the Background section to clarify the objective of our method and how it compares with existing methods. We also performed a side-by-side comparison between SMORE and two previous methods for spatial neighborhood analysis (HistoCAT and ImaCytE). These results are summarized in a new supplementary section and shown in Figure S4.

**- Demonstrating versatility of SMORE.** We performed spatial motif analysis on an atlas of mouse embryonic development generated by a sequencing-based technique, Slide-seq. This illustrates that SMORE, including its differential gene expression module, is not limited to imaging-based datasets and neural tissues. In this analysis, we also showcase clustering of tissues based on their spatial motif profile. The results are added as a new section to the manuscript (Section 3.7, Figures 9 and 10).

**- Demonstrating scalability of SMORE.** We applied our method to a whole mouse brain dataset (Allen Brain Cell atlas) containing about 4 million cells. The results are shown in a new supplementary section (Figure S7).

**- Performance details.** Following reviewer comments, we added a new table summarizing the timing performance of our method on experimental datasets (Table S1). We also included a supplementary section with recommendations for handling large sample sizes, which may arise when analyzing large datasets or sampling longer paths.

- **Abstract.** To comply with journal guidelines, we revised the abstract and made it more concise. In addition to these major changes, the revised manuscript includes several other updates and improvements. Below, we reply to all reviewer comments point-by-point. Reviewer comments are replicated in full in blue text with our responses provided in line in black *italics* text.

**Reviewer #1:**

The authors introduce a new approach to detect spatial motifs, representing recurring cell arrangements in tissues. Using synthetic and real data, the authors showcase the accuracy of their method in identifying these motifs, thereby unveiling novel insights into cell organization and gene expression in neural tissues. This approach has the potential to unveil tissue complexity, provide novel perspectives even in well-studied models, and generate testable hypotheses.

However, I have some concerns regarding the description and presentation of data in the manuscript.

Major concerns:

1. The manuscript lacks a detailed description of existing methods. In the Introduction section, the authors mentioned: "Therefore, existing methods for network motif analysis that focus on patterns of interactions between the nodes, rather than the identity of the nodes themselves, are not suitable for analysis of spatial data." In the Abstract section, the authors state: "most existing methods for analysis of spatial data only examine distribution of one or two labels at a time." However, the manuscript lacks detailed descriptions and examples of these "existing methods". It should provide comprehensive explanations of existing approaches, including examples, along with articulating the advantages of the new method over existing approaches.

***Response:*** *We appreciate this thoughtful comment and agree with the reviewer that detailed descriptions and examples of many existing methods were not included in the manuscript. In the original version of the manuscript, an overview of some existing methods was included in the first paragraph of the introduction (Page 2 lines 8-22), methods related to analysis of graphs were mentioned in the third paragraph of the introduction (Page 2 lines 41-54), and several other methods for analysis of spatial data were mentioned in the second paragraph of the Discussion section (Page 22 lines 51-60 and Page 23 lines 4-13). In light of this comment, and also a similar point raised by Reviewer #3, we modified the manuscript to (1) focus on methods that are more directly related to our work, i.e. analysis of spatial distribution of cell types, removing the mention of less relevant works that might cause confusion and (2) include a brief description and some examples of methods that are related to the present work together in one place (second paragraph of the Background in the revised version of the manuscript). Specifically related to this comment, we removed the emphasis on network motifs from the Background section, because the connection between our work and previous works on network motifs is subtle and technical. We now realize that emphasis on network motifs in the introduction section of the original manuscript was distracting and potentially confusing. We also want to acknowledge that there is a large diversity of methods for analysis of cell type distributions. Therefore, detailed description of all existing methods in this crowded and rapidly evolving field is more appropriate for a review article. We have cited two such articles in our revised manuscript [PMID: 34599004 and PMID: 36800999]. For this paper, our goal is to provide a conceptual overview of the existing methods that makes it easier to situate our work and compare it with existing methods. The advantage of our new method and the gap that it fills is now articulated in the third paragraph of the Background immediately following description and examples of existing methods.*

2. The advantages of the new method need to be further refined and substantiated. Indeed, whether the motifs discovered by this method are not identifiable by other approaches (such as those identifying niches in spatial data), requires further substantiation with data. Moreover, concise descriptions of the method's advantages over others and its biological value should be refined and included in the Introduction and Abstract sections.

***Response:*** *We thank the reviewer for this constructive feedback. The Background and Abstract are now updated to better communicate the advantages of our method and its biological relevance. Corresponding updates are included in the third and fourth paragraphs of the Background and also reflected in the revised abstract. Specifically, we want to highlight that, to our knowledge, SMORE is the only method that captures sequential arrangement or order of the cell types in the tissue. In contrast, methods that identify niches focus on regional composition of cell types, not sequential order. Identification of a motif associated with the primary rod pathway for scotopic vision (Fig. 4c-d) provides an example where cell type order is linked to biological function. Our method also includes an integrated differential gene expression analysis, which can provide biological insight as demonstrated in section 3.4 and Figure 5. We also agree with the reviewer that additional data is needed to substantiate whether the motifs identified by this method are distinct from those detectable by other approaches. Therefore, we applied two methods, HistoCAT and ImaCyte, on our bipolar dataset and compared the results with SMORE (Fig. S4). HistoCAT only considers pairwise interactions, whereas SMORE can analyze sequences of arbitrary length, which is a clear advantage. To directly compare the output of SMORE with HistoCAT, we focused on length-2 motifs. Our new analysis shows that SMORE length-2 motifs are generally consistent with HistoCAT results, but we also point out specific differences and discuss how these differences arise from emphasis on local cell type composition versus spatial order (Supplementary Information; Side-by-side comparison of SMORE with HistoCAT and ImaCytE; Paragraph 3). ImaCyte on the other hand searches for significant microenvironments or niches inside the tissue and doesn’t involve a specific length for the motif. ImaCyte output results included a motif with RBC and Type 2 (C) and Type 6 (K) cone bipolar cells (Fig. S4). This is similar to our most significant motif (“COOK”, Fig. 4c), although ImaCyte does not provide the spatial arrangement of these cell types that can connect them to the scotopic pathway, as shown in Figure 4d. Further, ImaCyte did not identify a motif similar to "HBBI" which, based on our gene expression analysis, can be linked to the function of type 1b OFF bipolar cells in the retina. Together, we believe this new analysis (1) clarifies how SMORE’s objective differs from methods that identify niches in spatial data, (2) demonstrates SMORE's ability to identify longer motifs, extending beyond length-2 motifs detected by tools like HistoCAT, (3) highlights SMORE’s sensitivity in uncovering patterns that may be missed by other methods, such as ImaCyte, and (4) illustrates how capturing sequential arrangements of cell types can add valuable biological context and aid biological interpretation of the motifs.*

3. The applicability to various tissues and cell types needs to be clarified. The manuscript primarily demonstrates the application of the method to spatial datasets composed mainly of neural cells (mouse retina and hypothalamus). Further clarification is needed regarding whether the method remains applicable to spatial data from other systems and cell types.

***Response:*** *To address this concern, as well as some of the following concerns, we analyzed spatial distribution of cell types in sections of mouse embryos at embryonic day (E) 8.5 and 9. This new analysis is added to the revised manuscript (Section 3.7, Figures 9 and 10). As acknowledged in the revised Background (Paragraph 3), we believe that sequential arrangements of cells are most relevant in the context of neural tissue. However, the framework of spatial motif analysis presented here is general and applicable to any spatial omics dataset. In less complex tissues and datasets, homotypic motifs may dominate the results. Nevertheless, as we show here, combining gene expression analysis with spatial motif discovery can reveal regional specializations in some cell types. Furthermore, we used this new analysis to demonstrate that spatial motifs can serve as structural tissue signatures, enabling clustering of tissues based on their spatial architecture.*

4. The applicability to various numbers of cell types needs to be clarified. The two datasets presented in this manuscript are from the mouse retina and hypothalamus, comprising over 100 and over 70 cell types, respectively. However, many spatial datasets do not contain as many cell types. In cases where there are fewer cell types, such as 10+ to 20+, does this method still apply? Further clarification is needed.

***Response:*** *The mouse retina dataset used in this study consists of 15 bipolar cell types. The confusion may have been caused by the first sentence of the first paragraph in section 3.3, which referred to all retinal cell types, not just the retinal bipolar cell types. We have modified the sentence to avoid confusion. In addition, the new analysis added in revision (Section 3.7) involves a dataset with 29 distinct cell states. In general, SMORE can be applied to datasets with arbitrary numbers of cell types.*

5. Further clarification is necessary regarding the application of this method on other sequencing platforms. The authors mentions in the Introduction section:"In contrast, new spatial techniques can profile the expression of many genes, or even the entire transcriptome. In this way, the position of many cell types can be mapped in the same sample. However, this feature is not directly utilized in virtually any of the existing analysis method…" All spatial transcriptomics data used in the manuscript are from -FISH sequencing platforms (SABER-FISH and MERSH), which have fewer genes compared to 10x Visium and Slide-seq technologies. Can this method be applied to 10x Visium and Slide-seq data? Additionally, further explanation is needed on how this method demonstrates the utilization of more genes.

***Response:*** *In the revised version, we included analysis of a mouse embryo dataset generated by Slide-seq, profiling the expression of 27,554 genes across more than 256,000 cells (Section 3.7). We demonstrated that SMORE can utilize full transcriptome level gene expression data in this dataset to identify genes that are differentially expressed in the spatial motifs (Fig. 10). Also, the revised version of the Background section, as discussed above, removes the emphasis on the number of genes profiled.*

6. Figure 3 demonstrates the performance on simulated data. However, how was this simulated data generated, and how overrepresented motifs were added? This process requires further clarification.

***Response:*** *In section 3.2, we describe how the synthetic data is generated and define the concept of embedding frequency for the synthetic data. In the revised manuscript, we included additional details in the Methods section 6.3 on “Applying SMORE on Synthetic and Real Data” to further clarify the embedding scheme.*

7. The manuscript lacks detailed descriptions of the methodology. For instance, in section 2.1.1, the author mentions: "…though this requirement is not necessary for directed graphs." The author utilizes Delaunay triangularization to construct a spatial graph, but how are directed graphs generated, and how is the direction determined? This may confuse readers. Similarly, the definition, significance, and references of ZNIC model and ZNIC counts should be further clarified.

***Response:*** *We appreciate the reviewer’s careful and thorough reading of the manuscript. The sentence in section 2.1.1 refers to the fact that URPEN can in principle also be applied to directed graphs. However, the current study only uses undirected graphs. So we have revised the sentence to avoid confusion. Significance is defined and described in detail in the Methods section on “Computing Significance”. We have now added a reference to it in the main text (Section 2.2, item 4). ZNIC model is also further clarified in the revised version (Section 2.2, item 3).*

8. In Figure 2d, the author evaluates the efficiency for k=4-8. However, in subsequent practical applications, the author mostly uses k=3. Therefore, the evaluation of efficiency in Figure 2 may not accurately reflect the real computational cost. It would be beneficial if the author could assess the computational cost on real data, including time and memory, as these are important considerations for users in practical usage.

***Response:*** *Although some examples of length 3 motifs (or k=3) are included in the manuscript, mainly for illustration purposes, most of the analysis in this paper involves motifs of length 4 and 5. For example, simulated graphs have been analyzed for motifs of length 4 (Fig. 3) and 5 (Fig. S3). Retina and hypothalamus datasets are analyzed for motifs of length 4 (Fig. 4 and 6). However, we agree that further assessment of computational cost can be beneficial. So, we have now included a table in the Supplementary Information (Table S1) detailing the method's performance in terms of timing for the real datasets analyzed in this manuscript.*

9. What impact do different choices of k have on performance and computational cost? Can the author assess this and provide recommendations to users?

***Response:*** *The number of possible paths increases exponentially with motif length. Despite this, URPEN remains highly efficient at sampling arbitrary length-k paths, as shown in Figure 2d. We have also optimized our MATLAB code for efficiency to handle large datasets, although memory issues are still possible when working with very large amounts of input data. Running times for k=4 and k=5 for retina and hypothalamus datasets are included in the new Table S1. We have also included a section in the Supplementary Information that provides recommendations to users working with larger sample sizes.*

Minor concerns:

1. In Figure 1a & 1c, please label the cell types for better reader comprehension.

***Response:*** *We have updated Figure 1a to address this concern. However, this figure, including panel 1c, does not refer to specific cell types. Instead, it aims to explain our method using simplified example scenarios. We revised the figure legend to clarify this point. Throughout the rest of the paper, letters are used to represent cell types and logos are used to illustrate spatial motifs. The correspondence between cell types and letters is detailed in the relevant figures that are based on experimental data (e.g. Figures 4, 6, and 8).*

2. In section 2.2, the author describes STREME as "…which the author has demonstrated to be more accurate, sensitive, and comprehensive than several widely used motif discovery algorithms." The task in this manuscript differs from that of the STREME article. Regarding whether STREME is also applicable to this task, could the manuscript provide some discussion? Additionally, STREME has only one author, so it should not be referred to using the plural "authors."

***Response:*** *We thank the reviewer for pointing out this error. The reference to the STREME author is fixed in the revised version. Although sequences of cell types sampled from neighborhood graphs are superficially similar to DNA or protein sequence, there are important differences. Therefore the STREME algorithm had to be modified substantially to account for these differences. Restriction on length of input sequence, the approach for generating shuffled control data, the strategy for counting motif instances, and the strategy for erasing nodes are all modified in SMORE. We describe the SMORE algorithm in section 2.2 and the Methods section. However, we avoid a direct, point-by-point comparison with STREME to maintain focus and avoid technical details that are not relevant to this study. Instead, we evaluate SMORE’s performance independently (Fig. 3 and S3), without assuming its suitability for this task solely due to its basis in STREME.*

**Reviewer #2:**

The authors propose a brand new concept of spatial motif, which is a very novel work that can help us to parse spatial transcriptome data from more perspectives. There are several problems to be solved by the authors as follows:

1. Please specify the "f" in figure 3 caption.

***Response:*** *We appreciate the reviewers feedback on the novelty and significance of the work. “f” refers to embedding frequency in Figure 3. This is now clarified in the figure caption.*

2. Please explain clearly the correspondence between "spatial motif" and "motif #1-5" in figure 6d/e.

***Response:*** *In Figure 6d-e, motifs are numbered from left to right. We have now clarified this in the figure and included more clarification in the caption.*

3. Due to unclear figure captions, it is difficult to fully grasp the author's intentions in figures 4b/c and figures 6d/e. Please provide additional details to complete the figure captions.

***Response:*** *The figure captions are revised for further clarity.*

4. Please elaborate on the differences between spatial motifs and the actual spatial locations of cell types. What are the advantages of using the spatial motifs for characterizing tissues?

***Response:*** *Spatial motifs are defined as a sequence of cell types, arranged in close proximity in a tissue, that occurs more frequently than expected by chance. As such, only a subset of each cell type may be arranged as a motif in the tissue. For example, in the insets of Figure 4c, cells that belong to one of top five spatial motifs are highlighted whereas other cells are shown in gray. Interestingly, we found gene expression differences between cells of the same type, depending on whether they are part of a motif or they are located elsewhere in the tissue. For example, Grm6 is downregulated in type 5b bipolar cells that occur in HBBI motif, compared to type 5b cells that are not part of this motif. We have revised the first paragraph of Section 3.4 to explain this more clearly. We have also updated the Background section (specifically paragraphs 3 and 4) to make the definition and the advantage of spatial motif analysis more clear. In addition to offering testable hypotheses about the functional significance of certain spatial arrangements, such as the mechanism proposed here for type 1b bipolar cells, the revised version also discusses the use of spatial motifs in tissue classification (Discussion; Paragraph 4). We illustrate this application with a new analysis of E8.5 and E9 mouse embryos (Section 3.7 and Figure 9).*

**Reviewer #3:**

The paper in itself is of interest, and the modeling of spatial configurations is the to go for this type of data; However, though the concept is powerful, and certainly of interest to the community, I have a number of major concerns: -I miss a number of highly relevant prior works that already did spatial cell motif modeling before spatial transcriptomics came about as a relatively new kid on the block. For instance, in Imaging Mass Cytometry, detailed cell type annotation followed by spatial configation modeling is commonplace for about a decade, and VeTRA and CODEX immunofluorescence tissue imaging idem dito. Software like histoCAT (Raul Catena), ImaCyte (Somarakis), and SpaceCo (again Somarakis) were specifically designed for detecting overrepresentation of cellular motifs in cohort comparison studies, and I think the related work should be reworked to include cell motif modeling in tissue regardless of the readout technology (spatial transcriptomics, Imaging Cytof or Imaging Mass Spectrometry). The presented approach in the current paper has indeed novel elements, but it is now presented as a first to look at complete cellular arrangements, and this claim should be toned down a lot. Original element I find the deployment of a motif discovery from genome sequences for detection of cellular motif overrepresentation, and that in itself is innovative enough.

***Response:*** *We thank the reviewer for their thoughtful and encouraging feedback, particularly their recognition of the interest in our work and the novelty of our approach. To address this concern, as well as a similar concern by reviewer #1, we have revised the Background section to better clarify the relationship between our method, which considers sequential order of cells, and previous methods, that focus on identifying spatial niches or microenvironments (Revised Background paragraphs 2 and 3). We have now also cited relevant methods that were developed using other types of spatial omics data, besides spatial transcriptomics, including HistoCAT and ImaCyte.*

-I find it difficult to distill the exact goal of the motif discovery: identify spatial motifs, but for what? Is the exact goal to find morif descriptors for a piece of tissue, or is it geared towards finding differences between patient groups (e.g. responders vs non-responders). This overarching goal should be articulated much more clearly.

***Response:*** *We believe that spatial motifs have both functional and structural implications. To better understand their functional significance, we conducted gene expression analyses on the cells that make up the motifs. In some instances, such as the "HBBI" motif in the retina, we can formulate hypotheses about the biological significance of that motif within the tissue. Structural significance of spatial motifs to applications like classification or grading of tissues was not directly mentioned in the original version of the manuscript. Inspired by this comment, we included an analysis in a new section on analysis of mouse embryonic tissues (section 3.7) which shows classification of tissue based on their motifs. We have also revised the Abstract, Background (Paragraph 3), and the Discussion section (Paragraph 3-4) to more clearly communicate these utilities of spatial motif analysis.*

-After introducing the necessity of motif discovery, I miss the exact motivation of the methodology that follows, and it read a bit like "let's go mine graphs with neighborhood info encoded". I totally support the presented method, but a bit more explicit motivation of the choice of methods would be beneficial for the paper clarity. Having said that, I find the method for sampling the graph original, and also subseqient motif discovery.

***Response:*** *We appreciate this comment and have updated the Background section to better describe the motivation for our methodology (revised Background paragraphs 3 and 4). Briefly, we were interested in examining patterns in sequential arrangement of cells rather than regional cell type compositions reflected in spatial niches, drawing inspiration from motifs in biological networks, such as gene regulation and protein interaction networks. Network motif discovery begins with an effective sampling method, which led us to develop URPEN for this purpose. The next step is to create a method for identifying significant patterns within these sequences, which prompted the development of SMORE in its current form. Additionally, gene expression analysis appears to be a natural extension of the motif discovery process.*

-I find the validation convincing, with first motif detection of artificially introduced motifs, and then a number of interesting real data findings. I also find the comparison of gene expression of cells of a specific type that are part of a motif in comparison to the same cell type not part of a motif highly informative. It would be interesting though to investigate whether the same motifs are recovered by HistoCAT of ImaCyte; with relatively minor effort this should be feasible.

***Response:*** *We have now included new analysis directly comparing our approach with HistoCAT and ImaCyte and provided a discussion about the similarities and differences between these methods in the Supplementary Information (Section on “Side-by-side comparison of SMORE with HistoCAT and ImaCytE’s performance on the retinal bipolar dataset” and Fig. S4). Please also see the response to concern 2 of reviewer #1.*

Final question: perhaps the authors can discuss the scalability to millions of cells: for instance the ABC-Atlas of spatial transcriptomics of a 3D whole mouse brain; Could this method be developed to develop a "spatial motif atlas of the whole brain?"

***Response:*** *URPEN and SMORE are both optimized for handling large datasets. We appreciate the reviewer’s specific suggestion for demonstrating the scalability of our method. In the revised manuscript, we included an analysis of the ABC-Atlas of spatial transcriptomics (Supplementary Information; section on “Scalability of SMORE: Analysis of a whole mouse brain spatial transcriptomics Atlas”, and Fig. S7). This dataset comprises approximately 4M cells across more than 5200 cell types. A 20% sampling of the graph generated from this dataset resulted in over 27 million samples. We successfully applied SMORE to this volume of data without enrichment which took about 15 hours on our computer to complete processing.*

Taken together, I find that this paper can certainly become of broad interest to others in the field, by addressing a number of the concerns I voiced. Though search for motif overrepresentation is not novel, the way that the authors address this is novel indeed.

***Response:*** *We thank the reviewer for recognizing the potential impact of our work and the novelty of our approach. We especially appreciate their valuable insights, and believe that incorporating these suggestions has significantly strengthened the clarity and overall quality of the paper.*

**2^nd^ round**

**Reviewer 1**

All my concerns have been addressed.

**Reviewer 2**

The authors did an excellent job in addressing all my comments, and I'm impressed by how well the method works in comparison to other methods, and on the ABC Atlas (which I'm very familiar with). The symmetry, striatal heterogeneity and scalability are now convincingly brought out. Also the context positioning, and comparison to state-of-the-art are now in much better shape: cudo's for running the two extra comparisons with HistoCat and Imacyte.

With the current revision, I definitely recomment acceptance of the manuscript. I have no suggestions for further improvement.
